# Synthesis and characterization of hybrid Anderson hexa­molybdoaluminates(III) functionalized with indometacin or cinnamic acid

**DOI:** 10.1107/S2053229618012536

**Published:** 2018-10-24

**Authors:** Nadiia I. Gumerova, Amir Blazevic, Tania Caldera Fraile, Alexander Roller, Gerald Giester, Annette Rompel

**Affiliations:** a Universität Wien, Fakultät für Chemie, Institut für Biophysikalische Chemie, Althanstrasse 14, Wien 1090, Austria; b Universität Wien, Facultät für Chemie, Zentrum für Röntgenstrukturanalyse, Währinger Strasse 42, Wien 1090, Austria; c Universität Wien, Institut für Mineralogie und Kristallographie, Althanstrasse 14, Wien 1090, Austria

**Keywords:** polyoxomolybdate, organic–inorganic hybrids, alk­oxy­lation, post-functionalization, hexa­molybdoaluminate, crystal structure, indometacin, cinnamic acid, anti­bacterial activity

## Abstract

The single-side Al-centred tris-functionalized hybrid organic–inorganic Anderson polyoxomolybdates (C_16_H_36_N)_3_[Al(OH)_3_Mo_6_O_18_(OCH_2_)_3_CNH(C_10_H_8_O)]·C_9_H_7_N·4CH_3_OH·5H_2_O (**AlMo_6_-NH-Cin**; Cin is cinnamic acid, C_10_H_9_O_2_) and (C_16_H_36_N)_3_[Al(OH)_3_Mo_6_O_18_(OCH_2_)_3_CNH(C_19_H_15_ClNO_3_)]·9H_2_O (**AlMo_6_-NH-Indo**; Indo is indometacin, C_19_H_16_ClNO_4_) have been prepared in a mild three-step synthesis and structurally characterized by single-crystal X-ray diffraction, ^1^H NMR and IR spectroscopies and elemental analysis. Both **AlMo_6_-NH-Cin** and **AlMo_6_-NH-Indo** crystallize in the ortho­rhom­bic space group *Pbca*. The anti­bacterial activities of **AlMo_6_-NH-Cin** and **AlMo_6_-NH-Indo** against the Gram-negative human mucosal pathogen *Moraxella catarrhalis* were investigated by determination of the minimum inhibitory concentration, which is 32 µg ml^−1^ for **AlMo_6_-NH-Cin** and 256 µg ml^−1^ for **AlMo_6_-NH-Indo**.

## Abstract 2

A post-functionalization protocol was used for the synthesis of two new tris-hybrid Al-centred Anderson-type polyoxomolybdates with indometacin or cinnamic acid.

## Introduction   

Polyoxometalates (POMs), an exceptional class of metal–oxide clusters with various compositions, exhibit an oxygen-rich surface with strong coordination potential (Pope, 1983 ▸). They have attracted much attention owing to their unique catalytic (Wang & Yang, 2015 ▸), redox (Gumerova & Rompel, 2018 ▸), magnetic (Clemente-Juan *et al.*, 2012 ▸) and bioactive properties (Bijelic & Rompel, 2015 ▸, 2017 ▸; Molitor *et al.*, 2017 ▸; Fu *et al.*, 2015 ▸; Bijelic *et al.*, 2018*a* ▸,*b* ▸) and constitute promising building blocks for advanced materials. Recently, increasing effort has been devoted to the introduction of organic and metal–organic units into the metal oxide frameworks in order to functionalize POM materials (Dolbecq *et al.*, 2010 ▸). Among the various synthetic strategies for the organic functionalization of POMs, alk­oxy­lation has gained much attention due to the diversity and tunability of alkoxyl ligands, especially when using the disk-shaped Anderson-type anions [*X^n^* ^+^H*_m_* *M*_6_O_24_]^(12–*n*–*m*)–^ (*M* = Mo^6+^ and W^6+^; *X* = heteroatom, *e.g.* Te^6+^ and I^7+^ for A-type with *m* = 0, or Al^3+^ and Ni^2+^ for B-type with *m* = 6), with a wide spectrum of central heteroatoms (Blazevic & Rompel, 2016 ▸; Zhang *et al.*, 2018 ▸). In particular, after Hasenknopf *et al.* (2002 ▸) had pioneered and established the synthesis of tris-derivatives of Anderson polyoxomolybdates (POMos), this archetype has been widely used as starting materials for the attachment of various tris [tris­(hy­droxy­meth­yl)methane]-based organic ligands [*R*C(CH_2_OH)_3_, de­noted R-Tris]. If the *R* group itself is reactive (–NH_2_, –CH_2_OH *etc*.), post-functionalization with a variety of organic mol­ecules, including ligands containing aromatic units (Al-Sayed *et al.*, 2015 ▸) or alkyl chains (Rosnes *et al.*, 2013 ▸) *via* imine, amide or ester-bond formation, is possible. The resulting hybrid materials were used in supra­molecular self-assembly (Macdonell *et al.*, 2015 ▸) or for the formation of metal–organic frameworks (MOFs; Li *et al.*, 2016 ▸). Major application fields are bio-inorganic (Yvon *et al.*, 2014 ▸), nano-structured (Song *et al.*, 2009 ▸), energy storage (Ji *et al.*, 2015 ▸), optical (Boulmier *et al.*, 2018 ▸) and photochemical (Schaming *et al.*, 2010 ▸) materials.

Herein, two biologically active mol­ecules, namely indometacin and cinnamic acid, were used to post-functionalize the Al-centred Anderson anion [Al(OH)_3_Mo_6_O_18_(OCH_2_)_3_CNH_2_]^3−^ (Wu *et al.*, 2011 ▸) *via* amidation reaction, resulting in two novel single-side grafted hybrid organic–inorganic Anderson-type POMos, namely (TBA)_3_[Al(OH)_3_Mo_6_O_18_(OCH_2_)_3_CNH(C_10_H_8_O)]·C_9_H_7_N·4CH_3_OH·5H_2_O (**AlMo_6_-NH-Cin**; Cin is cinnamic acid and TBA is tetra­butyl­ammonium) and (TBA)_3_[Al(OH)_3_Mo_6_O_18_(OCH_2_)_3_CNH(C_19_H_15_ClNO_3_)]·9H_2_O (**AlMo_6_-NH-Indo**; Indo is indometacin). Both com­pounds were structurally characterized by single-crystal X-ray diffraction, IR spectroscopy and elemental analysis. Their anti­bacterial activity against *Moraxella catarrhalis* was investigated by determination of the minimum inhibitory concentration (MIC).

## Experimental   

### Synthesis and crystallization   

#### Synthesis of AlMo_6_-NH-Indo   

Na_3_(H_2_O)_6_[Al(OH)_6_Mo_6_O_18_]·2H_2_O (**AlMo_6_**) was prepared according to a published procedure (Shivaiah & Das, 2005 ▸). The single-side attachment of Tris-NH_2_ to **AlMo_6_** was achieved through a modified published procedure (Wu *et al.*, 2011 ▸). **AlMo_6_** (3.84 g, 3.28 mmol) was dissolved in water (20.5 ml) and heated to reflux, when Tris-NH_2_ (0.735 g, 6.02 mmol) was added. After refluxing for 3 h, the solvent was removed by vacuum. The white powder obtained was redissolved with deionized H_2_O and then centrifuged to remove unreacted educts. Tetra­butyl­ammonium bromide (TBABr) (4.12 g, 12.8 mmol) was added to the solution and a white precipitate appeared. In order to functionalize **AlMo_6_-NH_2_** with indometacin, a mixture of indometacin (0.172 g, 0.500 mmol), **AlMo_6_-NH_2_** (1.05 g, 0.519 mmol) and EEDQ (*N*-eth­oxy­carbonyl-2-eth­oxy-1,2-di­hydro­quinoline; 0.143 g, 0.570 mmol) in CH_3_CN (9.00 ml) was stirred at 323 K for 24 h. The solvent was collected and removed by vacuum. The remaining yellow solid was redissolved in an MeOH–H_2_O mixture (2:1 *v*/*v*), followed by the addition of TBABr (0.5 g). After several weeks, crystals suitable for single-crystal X-ray diffraction were obtained [yield 2.3 g, 32% (based on Mo)]. FT–IR (cm^−1^): 324 (*s*), 368 (*s*), 395 (*m*), 442 (*s*), 484 (*s*), 505 (*m*), 534 (*m*, *sh*), 567 (*m*), 611 (*m*), 650 (*s*), 736 (*m*), 754 (*m*), 796 (*m*), 833 (*w*), 850 (*m*), 897 (*s*), 918 (*s*), 939 (*s*), 1012 (*w*, *sh*), 1027 (*m*), 1053 (*m*), 1072 (*m*), 1091 (*m*), 1122 (*m*), 1151 (*m*), 1174 (*w*), 1224 (*m*), 1290 (*w*), 1315 (*m*), 1336 (*w*, *sh*), 1361 (*m*), 1369 (*m*), 1396 (*sh*), 1458 (*m*), 1479 (*m*), 1552 (*m*), 1564 (*m*), 1610 (*m*), 1677 (*m*), 2871 (*m*), 2933 (*m*), 2960 (*m*), 3081 (*w*), 3322 (*m*, *br*). Elemental analysis for C_71_H_145_AlClMo_6_N_5_O_36_ (calculated) (%): C 38.38 (37.27), H 6.75 (6.61), N 3.06 (3.06), Cl 1.29 (1.55), O 22.3 (25.17). ^1^H NMR (500.32 MHz, CD_3_CN, 298 K): δ 0.96 (*t*, 36H), 1.34 (*m*, 24H), 1.59 (*m*, 24H), 3.53 (*m*, 24H), 3.62 (*s*, 2H), 3.81 (*s*, 3H), 2.15 (*s*, 3H), 6.66 (*dd*, 1H), 6.85 (*d*, 1H), 6.96 (*d*, 1H), 7.02 (*d*, 1H), 7.55 (*d*, 2H), 7.64 (*d*, 2H), 64 (*s*, 6H in CH_2_—μ_3_-O groups).

#### Synthesis of AlMo_6_-NH-Cin   

The preparation of **AlMo_6_-NH-Cin** was similar to that of **AlMo_6_-NH-Indo**, except that cinnamic acid (0.074 g, 0.500 mmol) was used instead of indometacin [yield 1.9 g, 27% (based on Mo)]. FT–IR (cm^−1^): 324 (*s*), 368 (*s*), 441 (*s*), 482 (*s*), 503 (*m*), 518 (*m*), 534 (*m*), 565 (*m*), 578 (*m*), 607 (*m*), 649 (*s*), 832 (*w*), 898 (*s*), 916 (*s*), 939 (*s*), 983 (*w*), 1035 (*m*), 1060 (*m*), 1112 (*w*), 1118 (*m*), 1153 (*m*), 1193 (*w*), 1228 (*m*), 1284 (*w*), 1323 (*w*), 1348 (*m*), 1380 (*m*), 1458 (*m*), 1479 (*m*), 1548 (*m*, *br*), 1575 (*w*), 1627 (*m*), 1668 (*m*), 1720 (*w*), 1731 (*w*, *sh*), 2874 (*m*), 2933 (*m*), 2960 (*m*), 3062 (*w*), 3290 (*m*, *br*), 3404 (*m*, *br*). Elemental analysis for C_73_H_154_AlMo_6_N_5_O_32.6_ (calculated) (%): C 36.48 (36.92), H 6.50 (7.00), N 2.92 (3.16), O 22.16 (23.10). ^1^H NMR (500.32 MHz, CD_3_CN, 298 K): δ 0.98 (*t*, 36H), 1.37 (*m*, 24H in TBA), 1.61 (*m*, 24H), 3.11 (*d*, 12H), 3.51 (*m*, 24H), 5.12 (*q*, 4H), 6.46 (*d*, 1H), 7.37 (*d*, 1H), 7.47–7.91 (*m*, 5H), 64 (*s*, 6H in CH_2_—μ_3_-O groups).

### IR spectroscopy   

Both compounds were identified by IR measurements on a Bruker Vertex70 IR Spectrometer equipped with a single-reflection diamond-ATR (attenuated total reflectance) unit in the range 4000–300 cm^−1^.

### ^1^H NMR   

NMR spectra were recorded on a Bruker FT–NMR Avance III 500 MHz instrument at 500.32 (^1^H) MHz in CD_3_CN at ambient temperature. Chemical shifts were referenced relative to the solvent signal for ^1^H nucleus.

### Elemental analysis   

The determination of C/H/N/O/Cl was carried out using an ‘EA 1108 CHNS-O’ elemental analyzer by Carlo Erba Instruments at the Mikroanalytisches Laboratorium, University of Vienna.

### MIC determination   

Minimum inhibitory concentrations (MICs) were determined by the broth microdilution method according to guidelines of the Clinical Laboratory Standards Institute (Wikler, 2009 ▸). Double dilutions of tested compounds in 96-well microtiter plates were prepared in the concentration range 1–256 µg ml^−1^. *M. catarrhalis* (ATCC 23246) was grown on Columbia agar with 5% defibrinated sheep blood. Inocula were prepared by the direct colony suspension method and plates were inoculated with 5 × 10^−4^ CFU per well. Results were determined by visual inspection after 20–22 h of incubation at 310 K in ambient air. Testing was performed by the standard broth microdilution method with azithromycin (Lode *et al.*, 1996 ▸) as the reference anti­biotic to assess test validity. MIC determinatiom was performed at the School of Medicine, University of Zagreb, Croatia.

### Refinement   

Crystal data, data collection and structure refinement details are summarized in Table 1 ▸. The positions of the independent H atoms were obtained by difference Fourier techniques and were refined with free isotropic displacement parameters. Fixed isotropic displacement parameters for all H atoms with a value equal to 1.5*U*_eq_ of the corresponding OH or H_2_O group atom were assigned. Restrained distances for *D*—H bonds were applied to avoid short *D*—H⋯H—*D* inter­actions. In the case of disordered groups, some bonds were added to or deleted from the connectivity array.

## Results and discussion   

**Figure 1:**
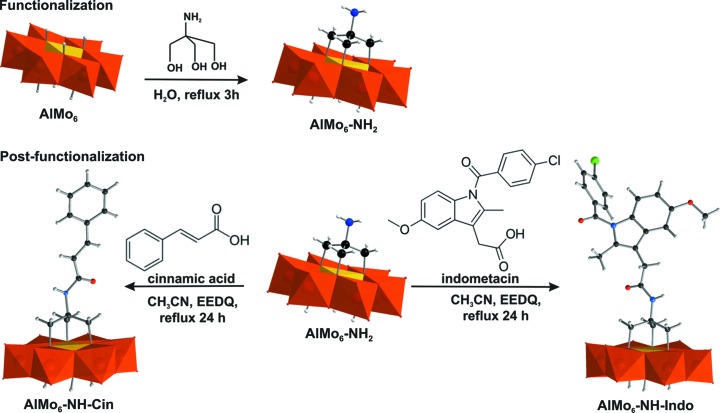
Functionalization of [Al(OH)_6_Mo_6_O_18_]^3−^ (**AlMo_6_**) with the Tris-NH_2_ ligand, followed by further post-functionalization of [Al(OH)_3_Mo_6_O_18_(OCH_2_)_3_CNH_2_]^3−^ (**AlMo_6_-NH_2_**) with indometacin or cinnamic acid, respectively. EEDQ is *N*-eth­oxy­carbonyl-2-eth­oxy-1,2-di­hydro­quinoline. Colour code: {MoO_6_} octa­hedra orange and {AlO_6_} octa­hedra yellow, with C atoms black, N blue, Cl green, H grey and O red.

**AlMo_6_-NH-Cin** and **AlMo_6_-NH-Indo** were prepared *via* post-functionalization by pre-forming the hybrid cluster **AlMo_6_-NH_2_** which was modified by amidation reactions (Fig. 1 ▸). The fact that single-side grafted anions were obtained supports an earlier theory claiming that the aqueous solvent is a key factor for the formation of single-sided Anderson derivatives (Wu *et al.*, 2011 ▸; Blazevic *et al.*, 2015 ▸; Gumerova *et al.*, 2016 ▸).

X-ray crystallographic analysis shows that the asymmetric units in **AlMo_6_-NH-Cin** and **AlMo_6_-NH-Indo** consist of the hybrid Anderson anion, three TBA counter-cations, solvent mol­ecules and, in the case of **AlMo_6_-NH-Cin**, one mol­ecule of quinoline as a by-product from EEDQ decomposition. The structural analysis revealed that both compounds crystallize in the ortho­rhom­bic space group *Pbca*. **AlMo_6_-NH-Cin** and **AlMo_6_-NH-Indo** both show the characteristic Anderson-type structure, with a central {AlO_6_} octa­hedron surrounded by six edge-shared {MoO_6_} octa­hedra that form a planar array of distorted octa­hedra (Fig. 1 ▸). Three different coordination modes of O atoms are found in the structure: six triple-bridged O atoms (denoted μ_3_-O) connect the heteroatom and two Mo atoms, six double-bridged O atoms (denoted μ_2_-O) connect two Mo atoms and two terminal O atoms (denoted O_t_) are connected to each of the six Mo atoms. The bond lengths of the three different binding modes are summarized in Table 2 ▸ and are in good agreement with other tris-functionalized Anderson POMos (Wu *et al.*, 2011 ▸; Al-Sayed *et al.*, 2015 ▸; Blazevic *et al.*, 2015 ▸).

The tris-ligand caps one side of the planar hexa­gon by binding to three μ_3_-O atoms of the {AlO_6_} fragment, whereas on the other side of **AlMo_6_-NH-Cin** and **AlMo_6_-NH-Indo**, the respective μ_3_-O atoms according to BVS calculations [−1.16 (O2), −1.20 (O4) and −1.19 (O6) for **AlMo_6_-NH-Indo**, and −1.15 (O1), −1.16 (O3) and −1.18 (O5) for **AlMo_6_-NH-Indo**, calculated according to (Brown & Altermatt, 1985 ▸)] are protonated.

**Figure 2:**
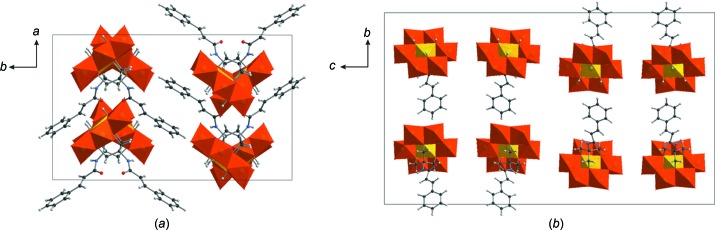
The crystal packing of **AlMo_6_-NH-Cin**, viewed along (*a*) the *c* axis and (*b*) the *a* axis. The TBA counter-cations and the solvent mol­ecules have been omitted for clarity. Colour code: {MoO_6_} octa­hedra orange and {AlO_6_} octa­hedra yellow, with C atoms black, N blue, H grey and O red.

The crystal packing of **AlMo_6_-NH-Cin** can be described as alternate layers of POMo anions and TBA counter-cations, which are repeated along the *c* axis (Fig. 2 ▸). The orientations of the hybrid polyanions along the *c* and *b* axes also alternate with an angle of approximately 85° between the planes of the inorganic Anderson ‘disks’ (Fig. 2 ▸ *a*). The attached ligands are turned towards each other along the *bc* plane. The distances between the inorganic POMo skeletons along the *a* axis are around 9.5 Å, and around 14 Å along the *b* axis. All four lattice water mol­ecules are situated in front of the undecorated side of the anion and form strong inter­molecular hydrogen bonds with μ_3_-O—H fragments, with short distances in the region 1.85–1.94 Å.

**Figure 3:**
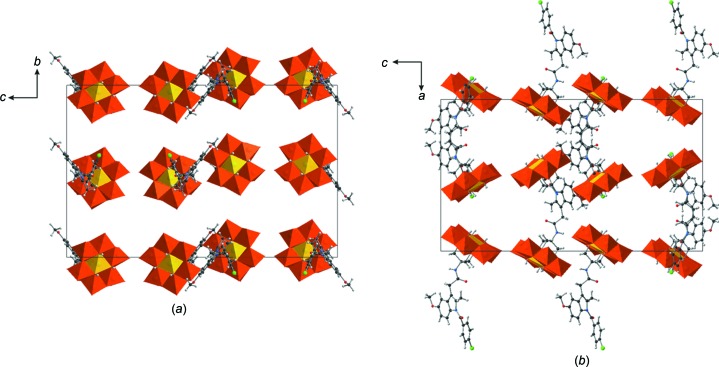
The crystal packing of **AlMo_6_-NH-Indo**, viewed along (*a*) the *a* axis and (*b*) the *b* axis. The TBA counter-cations and the solvent mol­ecules have been omitted for clarity. Colour code: {MoO_6_} octa­hedra orange and {AlO_6_} octa­hedra yellow, with C atoms black, N blue, H grey and O red.

The crystal packing of **AlMo_6_-NH-Indo** is similar to that of **AlMo_6_-NH-Cin** and can be described as alternate layers of POMo anions and TBA counter-cations, which are repeated along the *a* axis (Fig. 3 ▸). The orientation of the hybrid polyanions along the *c* and *b* axes is the same, with the grafted sides turned in different directions (Fig. 3 ▸ *b*). The distances between inorganic POMos along the *a* axis are around 12 Å, around 11 Å along the *b* axis and approximately 5 Å along the *c* axis. Six of nine lattice water mol­ecules are situated in front of the unfunctionalized side and form strong inter­molecular hydrogen bonds with μ_3_-O—H fragments and O_t_ atoms, with distances in the range 1.86–2.08 Å. The crystallographic refinement results for both **AlMo_6_-NH-Cin** and **AlMo_6_-NH-Indo** suggest no π–π inter­actions between the aromatic ring and the C=C double bond based on geometry and separation.

**Figure 4:**
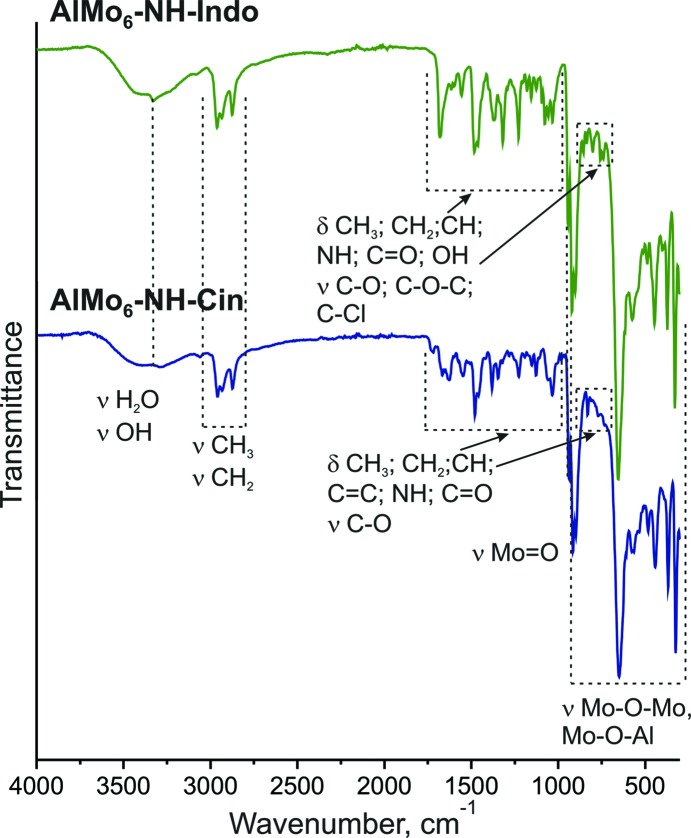
The IR spectra of **AlMo_6_-NH-Cin** and **AlMo_6_-NH-Indo** in the region from 4000 to 300 cm^−1^.

The IR spectra of **AlMo_6_-NH-Cin** and **AlMo_6_-NH-Indo** (Fig. 4 ▸) are typical for Anderson-type POMos and the characteristic peaks of the core structure are all in agreement with the peaks observed in the spectrum of Na_3_(H_2_O)_6_[Al(OH)_6_Mo_6_O_18_]·2H_2_O (Shivaiah & Das, 2005 ▸). The stretching vibrations of the terminal Mo=O units appear at 939 cm^−1^, whereas the peaks in the region from 300 to 920 cm^−1^ correspond to the anti­symmetric and symmetric deformation vibrations of the Mo—O—Mo and Mo—O—Al bridging fragments. The peaks appearing in the region 1030–1125 cm^−1^ could be assigned to C—O stretching vibrations, indicating the successful grafting of the tris ligands.

The anti­bacterial activities of **AlMo_6_-NH-Cin** and **AlMo_6_-NH-Indo** against the Gram-negative human mucosal pathogen *Moraxella catarrhalis* (Karalus & Campagnari, 2000 ▸) were investigated by determination of the minimum inhibitory concentration (MIC). **AlMo_6_-NH-Cin** shows a higher activity, with MIC values of 32 µg ml^−1^, while **AlMo_6_-NH-Indo** shows an MIC value of 256 µg ml^−1^. The MIC values for both compounds are much higher than for the clinically applied drug azithromycin, which has an MIC value of 0.06 µg ml^−1^. Taking into account that **AlMo_6_-NH-Cin** and **AlMo_6_-NH-Indo** have the same inorganic POMo part, counter-cations and net charge, it can be assumed that their anti­bacterial activities differs only due to the organic ligands attached. It is known that cinnamic acid and its derivatives exhibit anti­microbial activity against pathogenic and spoilage bacteria (Sova, 2012 ▸), indometacin in its turn, as a nonsteroidal anti-inflammatory drug (Lucas, 2016 ▸), showed bacteriostatic activity against *Helicobacter pylori* (Shirin *et al.*, 2006 ▸), whereas pure inorganic Ni- and Te-centred Anderson-type POMos and POTs are inactive (MIC > 256 µg ml^−1^) against *M. catarrhalis* (Gumerova *et al.*, 2018 ▸). Thereby, the activity of **AlMo_6_-NH-Cin** is caused by the synergistic effect of **AlMo_6_** and cinnamic acid, which is not the case for **AlMo_6_-NH-Indo**. The preliminary results obtained here show that not only does the activity of the attached ligand play a role, but also synergism with POMs strongly influences the properties of the hybrid compounds.

## Conclusion   

The success in synthesizing **AlMo_6_-NH-Cin** and **AlMo_6_-NH-Indo** shows the versatility and reproducibility of the post-functionalization protocol for the alk­oxy­lation of Anderson POMs. The attachment of bioactive ligands makes the hybrid Anderson POMos reported herein potentially superior to pure inorganic structures for anti­bacterial applications.

## Supplementary Material

Crystal structure: contains datablock(s) global, mo_ambl235_pbca, taco104_0m. DOI: 10.1107/S2053229618012536/jr3025sup1.cif

Structure factors: contains datablock(s) mo_ambl235_pbca. DOI: 10.1107/S2053229618012536/jr3025mo_ambl235_pbcasup4.hkl

Structure factors: contains datablock(s) taco104_0m. DOI: 10.1107/S2053229618012536/jr3025taco104_0msup3.hkl

CCDC references: 1847968, 1847969

## Figures and Tables

**Table 1:** Experimental details

	**AlMo_6_-NH-Cin**	**AlMo_6_-NH-Indo**
Crystal data		
Chemical formula	(C_16_H_36_N)_3_[Al(OH)_3_Mo_6_O_18_(OCH_2_)_3_CNH(C_10_H_8_O)]·C_9_H_7_N·4CH_3_OH·5H_2_O	(C_16_H_36_N)_3_[Al(OH)_3_Mo_6_O_18_(OCH_2_)_3_CNH(C_19_H_15_ClNO_3_)]·9H_2_O
*M*_r_	2227.19	2288.02
Crystal system, space group	Orthorhombic, *P* *b* *c* *a*	Orthorhombic, *P* *b* *c* *a*
Temperature (K)	100	200
*a*, *b*, *c* (Å)	16.1062 (17), 26.512 (3), 45.569 (5)	21.8904 (6), 23.9848 (6), 37.719 (1)
*V* (Å^3^)	19458 (3)	19803.9 (9)
*Z*	8	8
Radiation type	Mo *K*α	Mo *K*α
μ (mm^−1^)	0.84	0.85
Crystal size (mm)	0.23 × 0.15 × 0.03	0.15 × 0.12 × 0.05

Data collection		
Diffractometer	Bruker APEXII CCD	Bruker APEXII CCD
Absorption correction	Multi-scan (*SADABS*; Bruker, 2013 ▸)	Multi-scan (*SADABS*; Bruker, 2013 ▸)
*T*_min_, *T*_max_	0.666, 0.746	0.678, 0.746
No. of measured, independent and observed [*I* > 2σ(*I*)] reflections	293657, 17800, 15423	374956, 18115, 15743
*R*_int_	0.066	0.050
(sin θ/λ)_max_ (Å^−1^)	0.602	0.602

Refinement		
*R*[*F* ^2^ > 2σ(*F* ^2^)], *wR*(*F* ^2^), *S*	0.066, 0.144, 1.24	0.029, 0.075, 1.06
No. of reflections	17800	18115
No. of parameters	1129	1166
No. of restraints	39	53
H-atom treatment	H atoms treated by a mixture of independent and constrained refinement	H atoms treated by a mixture of independent and constrained refinement
Δρ_max_, Δρ_min_ (e Å^−3^)	1.27, −1.04	1.18, −0.64

Computer programs: *APEX2* (Bruker, 2013 ▸), *SAINT* (Bruker, 2013 ▸), *SHELXS97* (Sheldrick, 2008 ▸), *SHELXL2018* (Sheldrick, 2015 ▸) and *OLEX2* (Dolomanov *et al.*, 2009 ▸).

**Table 2:** Selected bond lengths (Å) in **AlMo_6_—NH-Cin** and **AlMo_6_—NH-Indo**

	**AlMo_6_—NH-Cin**	**AlMo_6_—NH-Indo**
Mo—μ_3_-O	2.291 (4)–2.391 (4)	2.3068 (19)–2.3632 (18)
Mo—μ_2_-O	1.910 (4)–1.941 (5)	1.912 (2)–1.944 (2)
Mo—O_t_	1.694 (5)–1.722 (5)	1.696 (2)–1.711 (2)
Al—μ_3_-O	1.864 (5)–1.923 (4)	1.863 (2)–1.927 (2)
